# Identifying the Non-Traditional Safety Risk Paths of Employees from Chinese International Construction Companies in Africa

**DOI:** 10.3390/ijerph18041990

**Published:** 2021-02-18

**Authors:** Chi Jin, Bo Li, Zhaoying Ye, Pengcheng Xiang

**Affiliations:** 1 Management in the Built Environment, Faculty of Architecture and the Built Environment, Delft University of Technology, Julianalaan 134, Delft 2628BL, The Netherlands; C.Jin-1@tudelft.nl; 2 School of Global and Public Affairs, IE University, Calle de Pinar 18, 28006 Madrid, Spain; Yozi1016@student.ie.edu; 3 School of Management Science & Real Estate, Chongqing University, Shazheng Street 174, Shapingba, Chongqing 400045, China; pcxiang@cqu.edu.cn

**Keywords:** safety risks, risk sources, risk paths, risk prevention, Chinese international construction companies, Africa

## Abstract

In recent years, more and more construction enterprises are expanding into overseas markets, especially in underdeveloped regions such as Africa. Compared to domestic construction projects, international construction projects have been faced with more uncertainties and increased levels of safety risks to the employees in the context of political turmoil, racism, and religious conflict in the host country. This study aims to answer what risk factors contribute to the threat to the safety of overseas employees and how safety risk factors interact, using employees from Chinese international construction companies (CICCs) in Africa as an example. A total of 39 safety risk factors were selected by literature review and case study based on Heinrich’s Domino Theory of Accident Causation. To identify the critical safety risk sources and significant risk paths, a questionnaire survey was conducted among 208 professionals who have participated in construction projects in Africa. Using structural equation modeling (SEM), a total of twelve critical risk paths and five controllable risk sources were identified. The improper behaviors of the CICCs and their employees were shown to have the largest impact on the safety of Chinese employees, through the mediating effect of the criminal offense. This study provides some insights into safety risk management in international construction projects. Meanwhile, the quantitative approach proposed can also be used by other international companies or governments in identifying the safety risk paths of their overseas workers involved in international construction projects.

## 1. Introduction

In recent years, research on international labor migration has received increasing attention due to its enormous social, economic, cultural, and public safety implications for both sending and receiving countries [1]. Multinational companies are sending employees overseas at an unprecedented rate. According to the International Labour Organization [2], the number of global international workers reached 164 million in 2017. The number of assignees has grown by 25% in the last decade and is expected to double by 2020 [3]. However, the safety of international workers has been under increasingly serious threat, especially in high-risk regions such as the Middle East, sub-Saharan Africa, and North Africa. A typical example is the 2013 In Amenas hostage crisis in which 36 foreign workers were killed before the military forces ended the incident. With the “ Arab Spring” sweeping through North Africa and the Middle East in 2011, companies and governments are faced with the challenge of ensuring the safety of their employees and citizens, whether through the provision of on-the-ground assistance or evacuation [4].

China has become one of the biggest international labor-sending countries [5]. The “Belt and Road Initiative” proposed by President Xi in 2013, focused on infrastructure construction has created great opportunities for Chinese international construction companies (CICCs) [6]. According to the Chinese Ministry of Commerce (CMC), the value of overseas newly-signed construction contracts grew steadily from USD 17.67 billion in 2003 to USD 241.8 billion in 2018 [7]. An increasing number of international construction projects have driven a significant number of Chinese employees from CICCs to engage in overseas markets. The number of new international workers for construction projects reached 227,000 in 2018, accounting for 46 percent of the total overseas workers [8]. Nevertheless, with more and more Chinese employees of CICCs going abroad, their safety has attracted extensive attention. According to the Ministry of Foreign Affairs (MFA) and the Embassies and Consulates abroad (ECA), the number of various security cases has climbed from 30,000 in 2003 to around 79,000 in 2018 [9,10]. The safety problem is especially severe in Africa. According to incomplete statistics by the Ministry of Foreign Affairs in 2015, the occurrence rate of security incidents involving Chinese personnel in Africa was almost four times higher than that in the other continents [11]. Compared with traditional safety incidents caused by unsafe working conditions or misoperation of the workers during the construction process, such as electrical shock or falling from a height [12,13], the non-traditional safety risk such as kidnapping, armed robbery, and dangerous pandemics are even more severe and frequent [14]. For example, in 2015 three senior executives of China Railway Construction died in a terrorist attack in Mali, Africa. In 2017, a Chinese construction site in the Democratic Republic of the Congo was robbed by armed criminals, which caused five Chinese injured (see Appendix A (Table A1) for more security cases involving the Chinese employees from CICCs). Therefore, in this paper, we only focus on non-traditional safety risk factors for Chinese employees from CICCs in Africa. 

The severe safety problem of Chinese employees from CICCs in Africa can be so far explained with four main reasons. First, some countries in Africa are experiencing turbulent issues such as political instability, social insecurity, and frequent outbreaks of epidemics [15]. Second, most of the construction project sites are located in remote and sparsely populated areas, making them more likely to be targeted by criminals [16]. Besides, vast ethnic, religious, or cultural differences between the Chinese and the locals often lead to continuous conflicts [4]. Last but not least, Chinese construction employees’ security awareness and emergency response capacity are relatively low. 

How to ensure the safety of employees from CICCs has become an important topic in both academia and practice. Many researchers have tried to tackle the problem from the risk perspective. For example, Venter [17] maintained that exposure to increased levels of security risks emphasized the importance of corporations’ ability to manage risk effectively. According to Noland [18], popular attitudes towards globalization may signal a degree of security risk, which refers to the possibility that facilities may be subject to sabotage and staff to harassment or assault. A lot of research has been conducted to identify various types of risks in international construction projects, but most of them mainly focus on the project itself such as financial risks, exchange rate risks, and contract risks instead of the safety of the personnel [19,20,21,22]. Although some researchers studied personal safety risks to Chinese overseas citizens such as overseas students and tourists [23,24,25], few studies have focused on the employees from CICCs, who are faced with more serious and complex safety risks given the long-term duration of construction projects and the complex interests involved [26,27]. Besides, many studies have treated safety risk factors as individual factors without exploring the links and differences between them, which may underestimate the severity of certain risk factors [16,17]. According to Cendrowski and Mair [28], risks should not be segmented and managed independently because safety risks are dynamic, fluid, and highly interactive. Therefore, it is important to classify the safety risk factors and regard it as a dynamic process during risk analysis and management. 

The purpose of this research is to identify the non-traditional safety risk sources and risk paths of international workers in the construction industry and to propose an alternative safety risk prevention strategy, using employees from CICCs in Africa as an example. Although this study focuses on Chinese employees in Africa, a similar approach can also be used by international companies or governments from other countries in identifying the safety risk paths of their overseas workers in international construction projects.

The remainder of the paper is structured as follows. In the subsequent section, we review some selected literature on the non-traditional safety risk management approach and Heinrich’s Domino Theory of Accident Causation, followed by the classification of the safety risk factors. Then we propose a safety risk path model based on the results of the literature review and case study. Next, we introduce an empirical research design, the data collection process, and the statistical methods. After that, the results of the empirical study are presented and discussed. The paper concludes with a review of the key findings and limitations.

## 2. Literature Review

### 2.1. Non-Traditional Safety Risk Management Approach

Since Fayard introduced the concept of risk management in 1931, risk management has evolved into a systematic approach following a risk identification–risk assessment–risk response–risk monitor loop [29,30]. Verbano and Venturini [31] proposed nine areas of risk management, including enterprise risk management [32], project risk management [33], and disaster risk management [34], etc. Although safety risk management is not listed as one of the above nine areas, it is an important component in almost every area. According to the British Health and Safety Commission [35], safety management concerns health and safety performance and legal compliance, as well as loss control. In practice, the safety risk management of overseas citizens has been carried out mainly at the national, corporate, and individual levels.

A common safety risk management approach at the national level is to establish a special department or website, led by the foreign ministry or consulate of the source country, to provide companies and individuals abroad with risk advisory information such as risk warning maps, risk reaction guides, etc. [4]. For example, the UK Foreign and Commonwealth Office (FCO) explicitly advises businesses in high-risk areas by establishing the Overseas Business Risk (OBR) service, which provides country-specific risk data on terrorism, political terrain, crime, etc. [36]. A coalition of nine governments, including the United States, the United Kingdom, the Netherlands, and Norway, has agreed to help implement the Voluntary Principles on Security and Human Rights, which is a public-private risk assessment partnership that enables international companies to avoid steps in their risk management processes that could inflame local tensions and thereby reduce security risks to nationals abroad and protect human rights [4]. There have been various research studies on security risk management for different companies. For example, Zumkehr [37] developed a Risk Management Expense Portfolio Tool to help companies estimate the cost of security risk management. He also stated that the process of employee safety risk management consists of three elements: risk preparation (e.g., insurance, risk assessment), risk response (e.g., crisis management, program discontinuation), and prevention of initial or ongoing loss or injury (e.g., psychological support services for employees). Claus [38] developed an integrated risk management model, which has eight steps in accordance with the “Plan-Do-Check” cycle. Using information from 628 companies and 718 respondents worldwide, he assessed the threats in 20 different industries and came up with a geographical risk map for international companies to ensure the safety of their employees. Regarding individual security risks, A number of studies have investigated the safety of different groups abroad, for instance, international students and tourists [39,40]. Scholars have summarized the security risk checklists for citizens abroad included terrorist attacks, kidnappings, hijackings, extortion, and robberies [41,42]. In addition, researchers explored how to manage safety risks using employee behavior change approaches and employee risk perception methods [43,44,45]. 

Most of the above-mentioned studies on safety risk management for overseas employees have used a risk checklist approach for the identification of safety risks. The advantage of the risk checklist approach is that risk managers can construct risk checklists that are specific to different companies and regions and continually adjust them [46]. However, the risk checklist approach regards risk factors as independent variables and ignores the interactions between them, which may lead to biased results. 

### 2.2. Interaction among Risk Factors

According to Heinrich’s Domino Theory of Accident Causation [47], social and physical environment will lead to fault or carelessness of a person, which will further result in unsafe acts or conditions, that cause accidents and subsequent injuries. These five standing dominos (i.e., social and physical environment, fault of a person, unsafe acts and conditions, accidents, and injuries) form Heinrich’s domino theory, which would fall one after the other if the first domino falls, and the accident can be avoided only if the sequence chain is disrupted [48]. The theory was later extensively used in the field of safety risk management by some researchers [49,50]. In most cases, safety risk factors are divided into three groups, namely risk sources, risk events, and risk consequences [51]. Risk sources lead to risk events, which further lead to risk consequences, forming a so-called risk path [52,53]. If the risk path is disrupted (by breaking the connection between risk sources and risk events or between risk events to risk consequences), the risk consequences will not occur. Therefore, identifying and breaking the critical risk paths can contribute to effective security risk prevention [53].

Risk consequences are typically defined as the impact of risk factors on project goals such as expense, time, efficiency, customer satisfaction, and safety [54]. Since we focus on the safety of employees from CICCs, only two risk consequences are generally considered, i.e., casualties and property loss (Property loss is often related to the security of a person in practice [11]. For example, assume that one was robbed and (s)he handed over money to survive. Although there is no casualty in this case, property damage is caused). A risk event is the occurrence of an undesirable incident such as robbery and collapse of a building [51]. Risk sources are characterized as elements that alone or in combination have the intrinsic potential to give rise to risk events such as natural disaster, break of war, political unrest, and tribal clashes [55]. Notably, researchers often further classify risk sources. For example, Eybpoosh et al. [51] classified the risk sources of international projects into unexpected situations and adverse changes. In this research, we classify the safety risk sources into controllable risk sources (e.g., lack of safety awareness) and uncontrollable risk sources (e.g., outbreak of war) because our purpose is to prevent the spread of the risk sources, thus avoid the occurrence of the risk consequences. 

**Figure 1:**
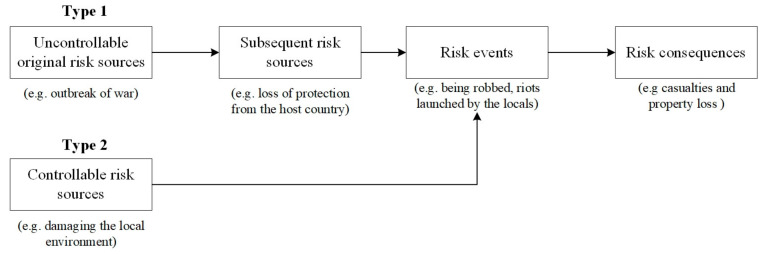
The safety risk path model for employees from Chinese international construction companies (CICCs) in Africa.

In hazard research, if the disaster cannot be directly controlled, its controllable subsequent disaster will be explored [56]. Therefore, we borrow the concept “subsequent disaster” from hazard theory, where we cannot directly control the uncontrollable risk source leading to a risk event, we further explore its subsequent risk source caused by the uncontrollable risk source and avoid the risk event by controlling the subsequent risk sources. In other words, for controllable original risk sources, we consider they are directly linked to risk events. Whereas for uncontrollable original risk sources, we further identify the subsequent controllable risk sources in order to prevent the spread of risk sources. The two types of safety risk path models are depicted in Figure 1. In the first type, risk sources cannot be directly avoided (e.g., break of war, political instability). Therefore, we further identify its subsequent controllable risk sources (e.g., loss of protection from the host country). In the absence of host country protection, we can guarantee the safety of personnel by purchasing security from a third-party company. However, the controllable risk sources (type 2) can be directly avoided. For instance, “damaging the local environment” can be avoided by taking environmental initiatives.

### 2.3. Potential Safety Risk Factors of Employees from CICCs in Africa

Researchers have identified a number of risks threatening the safety of overseas Chinese workers in the construction industry. For example, Ullah et al. [57] found the political risks such as conflicts and wars can not only affect the success of projects but also pose a great threat to the personal safety of Chinese employees in Africa. Du [58] maintained that besides political instability, economic risks, and corruption in the host country, security risks also arise from the misbehavior of employees themselves. Chen and Orr [15] argued that the main security threats for CICCs in Africa are insurgencies, racial conflicts, and certain diseases that are rare or non-existent in China, such as AIDS, malaria, cholera, and typhus. Chen et al. [59] found misconceptions and stereotypes between Chinese and Africans might lead to discrimination and conflict and threaten the safety of employees from CICCs in Africa.

Based on the above literature review, it is clear that the safety risks of employees from CICCs in Africa can be classified into risk sources (including uncontrollable original risk sources, subsequent risk sources, and controllable risk sources), risk events, and risk consequences. In this section, we will identify the safety risk factors and classify them into those categories based on extensive literature review and case study. The literature review is a frequently used method for identifying risk factors [60]. Articles considered in the literature review were related to the safety risks of Chinese employees in Africa and published in international and Chinese scientific journals up to November 2020. As a supplement, the cases in Appendix A (Table A1) also serve as a reference for risk factors. Finally, we identified 27 risk source factors including original and subsequent risk sources. These risk sources were classified into five groups, namely political, economic, sociocultural, environmental, and behavioral risk source factors. We consider original political, economic, sociocultural, and environmental risk sources to be uncontrollable original risk sources or external risk sources, which might lead to subsequent risk sources. Whereas the improper behavior of CICCs and their Chinese employees are controllable risk sources or internal risk sources. Furthermore, we identified 10 risk events in three categories, which were armed conflict, criminal offense, and accidental injury. Risk consequences were categorized into casualties and property loss. The specified risk factors and sources of reference are shown in Table 1.

## 3. Methodology 

### 3.1. Research Design 

**Figure 2:**
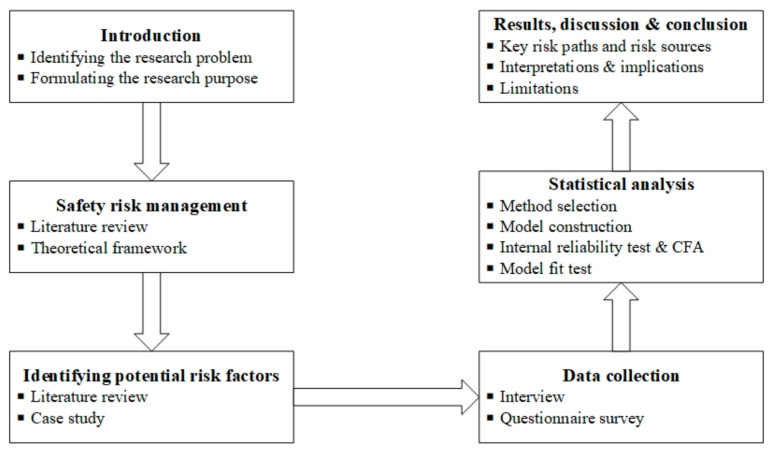
The methodology of this research.

This research aims to identify the critical non-traditional safety risk sources and risk paths of international workers in the construction industry, using employees from CICCs in Africa as an example. To achieve this purpose, both qualitative (potential risk factors) and quantitative (scoring of these risk factors) data are needed. In the previous section, we identified the potential safety risk factors and risk paths of employees from CICCs by literature review and case study. To verify whether these risk paths exist in practice and explore which risk paths are more critical, empirical research needs to be conducted based on a questionnaire survey. Figure 2 presents the methodology of this research.

### 3.2. Data Collection

A questionnaire form was constructed consisting of two main parts. Following Irem et al. [52], the first part is about the basic information of the respondents while the second part asked the respondents to rate the severity of the risk factors through a five-point Likert scale ranging from 1 “very low” to 5 “very high”. Respondents with work experiences on international construction projects in Africa were reached through face-to-face meetings or via email from September to December in 2017. Since there were little data about how CICCs and their employees were distributed in Africa, the snowball sampling method was used. Specifically, after completing the questionnaire, the respondents were asked to forward the questionnaire to his/her acquaintances who were also working in CICCs in Africa or having previous work experiences. In this way, we can obtain a sufficient number of samples. A total of 227 respondents completed the questionnaire while 19 questionnaires were invalid due to missing data. Finally, the survey generated 208 valid questionnaires. The characteristics of the respondents were shown in Table 2. 

### 3.3. Structural Equation Modeling 

Several data analysis methods can be used to empirically analyze the relationship between variables, such as regression analysis and path analysis. However, regression analysis cannot deal with the case where there is more than one dependent variable, nor can it deal with the multicollinearity among independent variables. Although simple path analysis can examine the interrelationship between independent variables, it cannot examine the relationship between dependent variables as a whole. Therefore, a complicated path analysis—Structural Equation Modeling (SEM)—is employed. SEM is a comprehensive statistical approach to test hypotheses about relations among related factors [69]. SEM allows complex variable relationships to be described through hierarchical or non-hierarchical, recursive or non-recursive structural modeling, to present a more complete view of the entire model [70]. SEM can also be used to estimate various causal relationships between variables and calculate all paths simultaneously, namely direct (C→R), indirect (C→M→R), and multiple relationships (C→M→R, M→R→O) [71,72]. Considering the hierarchical structure and intricate paths of the safety risk, SEM was selected for empirical analysis in this study.

According to the general rules of SEM that the sample size should be greater than 100, preferably greater than 200 [73,74], our sample size of 208 is adequate. The commonly used method to estimate coefficients in SEM is the maximum likelihood (ML) [75]. A preliminary analysis of the collected data showed that the data were normally distributed because the z-score values of kurtosis and skewness were within (−1.83, 0.197) and (−1.64, 1.95), which supported the utilization of the maximum likelihood method for parameter estimation in this study (Generally, if the z-score value of the kurtosis and skewness of the variables are within −1.96 to 1.96, the data are normally distributed). However, it does not report the significance of the indirect effects and total effects. Therefore, the bootstrap technique built-in AMOS was also adopted to compute the confidence intervals for the indirect and total effects [76]. 

To test whether the data fit the model well, different fit indexes should be evaluated which address different aspects of model appropriateness (e.g., parsimony, sample size effects, comparisons to null models) [77]. In this paper, four distinct indexes were selected for evaluation of the model fit and its suitability, which were comparative fit index (CFI), root mean square error of approximation (RMSEA), parsimony normed fit index (PNFI), and square to df ratio (CMIN/DF). CFI > 0.90, RMSEA < 0.08, PNFI > 0.50 and CMIN/DF < 5 show an acceptable model fit [78].

#### 3.3.1. Measurement Model

The construction of a measurement model is the first step for SEM analysis. Two types of variables are involved in SEM: observed variables and latent variables. The former can be evaluated explicitly, while the latter is abstract or theoretical constructs derived from the observed variables [51]. The measurement model is the component of the model that discusses the relationship between the latent variables (e.g., uncontrollable original political risk sources) and their measurement variables (e.g., outbreak of war, political unrest, and government corruption). In the present paper, we used Cronbach’s alpha and CFA to test the reliability and validity of our data.

##### Internal Reliability 

Cronbach’s alpha(α) coefficient is a commonly used way to measure the internal reliability of the measurement model. Generally, when the α coefficient is higher than 0.7, it can be considered that the internal reliability of each latent variable is high and meets the requirements [79]. Using SPSS reliability analysis, the various latent variables reliability results obtained are shown in the sixth column of Table 3. The Cronbach alpha of every latent variable was acceptable.

##### Confirmatory Factor Analysis (CFA)

Confirmatory factor analysis (CFA) is an important tool that uses actual collected data to identify the validity of the measurement models [80]. Convergent validity and discriminate validity are two main indicators of validity. Convergent validity explains the degree of correlation and shared variance among the observed variables of a latent variable [71]. In general, when Average Variance Extracted (AVE) > 0.5 and Factor loading > 0.4, convergent validity is of the required standard [71]. Discriminant validity is a metric that depicts to what extent a latent variable differs from other latent variables [71]. When the biggest squared intercorrelation between latent variables (BSIBLV) < AVE, it means that the discrimination validity is up to standard. The CFA results showed that the factor loading of GAA (getting into an automobile accident) was 0.265, which was below 0.40. Therefore, the risk event GAA was removed. Table 3 shows the convergent validity and discriminate validity of every latent variable.

#### 3.3.2. Structural Model 

**Figure 3:**
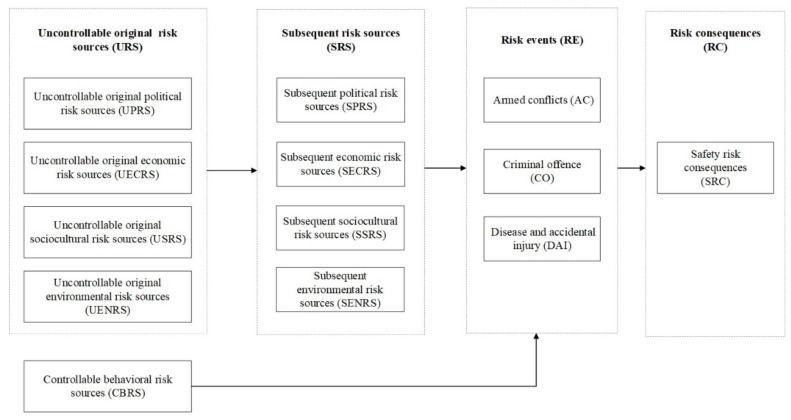
The structural model of safety risk for employees from CICCs in Africa.

The structural model is the relationship between the latent variables. In this section, we develop the structural model based on the risk path model in Section 2.2. As demonstrated before, risk source factors should be distinguished into uncontrollable and controllable sources to investigate its further spread. The structural model of this study is presented in Figure 3.

In this model, the uncontrollable original risk sources and controllable risk sources are exogenous variables, which means they are determined outside the model and are imposed on the model. Whereas the subsequent risk sources, risk events, and risk consequences are endogenous variables, whose values are determined by the model.

## 4. Results

**Figure 4:**
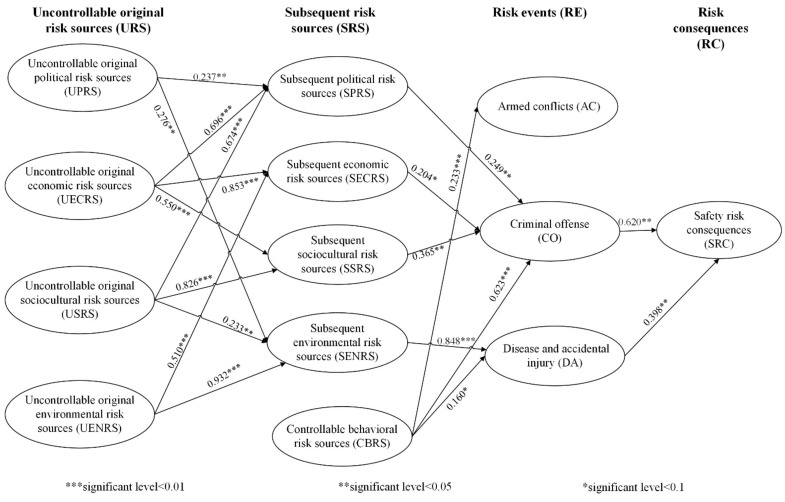
Final risk path model. Model fit: CFI = 0.905; RMSEA = 0.051; PNFI = 0.722; CMIN/DF = 2.341

The purpose of this research is to identify the critical safety risk sources and risk paths of employees from CICCs in Africa. Figure 4 presents the safety risk spread network and standardized coefficients of risk paths. The risk paths illustrated in Figure 4 are all significant paths; insignificant risk paths are not presented. The goodness-of-fit measures support the adequacy of the model. 

### 4.1. Identification of Key Risk Paths 

Table 4 shows the path analysis results. A total of 12 risk paths were statistically significant. We consider these 12 risk paths to be the critical risk paths for this study. We can also find that there is a strong causal relationship between uncontrollable original risk sources (URS) and subsequent risk sources (SRS), except for a few casual relationships that are not significant, and that URS leads to the occurrence of risk events through the mediation effects of SRS. For instance, uncontrollable original sociocultural risk sources (USRS) lead to subsequent environmental risk sources (SENRS), which further result in disease and accidental injury (DAI). Surprisingly, no significant relationship was found between subsequent risk sources and armed conflicts (AC), and AC was not significantly related to risk consequences. In contrast, the criminal offense (CO) was strongly related to safety risk consequences.

We further calculate the risk paths’ coefficient to assess the relative importance of the risk path. According to the basic principles of path analysis in SEM [81], the total effect of the entire risk path is estimated as the product of the individual coefficients for each direct effect that makes up that causal path. For example, the total causal effect coefficient of path UPRS → SPRS → CO → SRC is obtained by multiplying the coefficients of the three paths UPRS → SPRS, SPRS → CO, and CO → SRC. The results are presented in the last column of Table 4. We can see that the path with the largest total effect coefficient is CBRS → DAI → SRC (0.386), followed by UENRS → SENRS → DAI → SRC (0.315) and UECRS → SSRS → CO → SRC (0.187). The path with the smallest total effect coefficient is UPRS → SPRS → CO → SRC (0.037).

### 4.2. Identification of Key Risk Sources 

As demonstrated before, the risk consequences can be avoided by control the spread of risk sources. For controllable risk sources, their spread can be controlled directly while the uncontrollable risk sources cannot be controlled directly. For them, we should further identify and control their subsequent risk sources, thus preventing the risk spread process. Therefore, the key risk sources that we ultimately need to identify are the key controllable risk sources and the subsequent risk sources.

Table 5 presents the results of total effects and significance of controllable risk sources and subsequent risk sources calculated by AMOS. We found that five risk sources had a significant impact on the security risk consequences, which are the key risk sources. Further discussion of these results will be presented in the next section.

## 5. Discussion

The results of our empirical study showed that the safety risk factors of employees from CICCs in Africa interacted with each other and thus form a dynamic safety risk network, which corresponded with previous research [26,51,53]. This implies that we should consider the interrelationship between risk factors when carrying out risk studies in order to avoid underestimating the severity of risk by examining only individual risk factors. 

A total number of 12 critical safety risk paths were identified. The risk path with the largest path coefficient is “Controllable behavioral risk sources (CBRS) → Criminal offense (CO) → Safety risk consequences (SRC)”. Besides, CBRS had the greatest impact on safety risk consequences. This result corresponds with Heinrich’s Domino Theory of Accident Causation which emphasizes the importance of unsafe behavior of employees and enterprises. It is understandable since more and more conflicts result from the inappropriate behavior of Chinese employees and construction enterprises. For example, Corkin [65] pointed out that Chinese companies in Africa have a reputation for being reluctant to hire local labor, preferring to bring labor from China, which causes discontent among job-seeking Angolans. In 2016, about 200 local youths expressed their discontent over not being able to share employment opportunities and injured 14 Chinese workers on a Chinese-funded railroad project in West Narok County, Kenya (case 10). 

The risk path with the second-largest path coefficient is “Uncontrollable original environmental risk sources (UENRS) → Subsequent environmental risk sources (SENRS) → Disease and accidental injury (DAI) → Safety risk consequences (SRC)”. Coincidentally, the SENRS had the second-largest impact on safety risk consequences. Compared to other countries, one of the distinctive features of the African region is the relatively harsh living environment. Most Chinese construction projects in Africa are located in rather remote areas. In the numerous reports of security incidents of Chinese employees in Africa, we can see that crimes like attacks on project camps, robberies, and thefts occur frequently and further lead to casualties and property loss if the construction project is exposed to insecure conditions. Besides, Chinese employees from CICCs are prone to suffer natural disasters like landslides and infectious diseases like malaria, which is consistent with Cervellati et al. [82]. 

The next important risk path is “Uncontrollable original sociocultural risk sources (USRS) → Subsequent sociocultural risk source (SSRS) → Criminal offense (CO) → Safety risk consequences (SRC)”. The huge cultural difference between China and Africa has exacerbated the conflicts and tensions between Chinese employees and the locals. Corkin [65] has found that many Chinese and Angolan interviewees showed their contempt for each other. Except for the lack of local labor, the locals believe that Chinese employees are taking away their wealth. This phenomenon also happened during our interview with Chinese employees. For example, some interviewees expressed discriminatory perceptions towards local Africans during our interview. This racial stereotype is also well documented in the research of Ching [83]. This two-way racism between Chinese employees and locals often ends up with Chinese employees being robbed, stolen, or injured.

Concerning risk events, Criminal offense (CO) has the largest impact on risk consequences, followed by Disease and accidental injury (DAI). This finding is consistent with Tao et al. [14], who highlighted the potential threat to the personal safety of Chinese people posed by the frequent occurrence of security problems such as robbery, kidnapping, theft, and extortion. Interestingly, we found armed conflicts (AC) were not significantly related to safety risk consequences. This may be because the Chinese government takes such risk events very seriously and discourages construction companies from going to these dangerous areas for construction projects. Previous research has pointed out that breaking risk chains in risk networks is an important tool for risk prevention [26]. In this research, to prevent the personal safety risks of Chinese employees of CICCs in Africa, certain measures need to be taken to hinder the two risk paths of “risk sources → risk events” and “risk events→risk consequences”. Using the risk path “Controllable behavioral risk sources (CBRS) → Criminal offense (CO) → Safety risk consequences (SRC)” as an example, our safety risk prevention can be split into two steps. First, we can prevent the safety risk from spreading from CBRS to CO. In specific, CICCs and their Chinese employees can improve the local people’s perception of them through their practical initiatives such as reducing the damage to the local environment caused by the construction process, providing more employment opportunities for the locals, and enhancing communication between Chinese employees and the locals. The second step is to block the spread of safety risk from CO to SRC. As suggested by Tao et al. [14], CICCs can establish a safety management system, which includes physical security facilities, a pre-warning system, etc. Moreover, professional security personnel can be hired to protect the construction site and employees if necessary.

## 6. Conclusions

The purposes of this study are to identify the key safety risk paths of Chinese employees from CICCs in Africa and identify the key controllable safety risk sources. Based on Heinrich’s Domino Theory of Accident Causation, we constructed a theoretical model of security risk for Chinese employees from CICCs in Africa. Furthermore, we used structural equation modeling to analyze 208 questionnaires collected from employees working in CICCs in Africa or having related work experiences. In sum, 12 critical safety risk paths and 5 key controllable safety risk sources were identified. The identification of significant risk paths demonstrates the existence of interrelationships between safety risk factors and the applicability of Heinrich’s Domino Theory of Accident Causation. We found that the inappropriate behavior of CICCs and their employees is most associated with the occurrence of safety risk consequences, followed by subsequent environmental risk sources and subsequent sociocultural risk sources.

Different from previous studies that only qualitatively identified safety risk factors, the present paper emphasizes the heterogeneity of risk sources and explores the interactions between safety risk factors. This study enables a better understanding of how the safety risks are transmitted in the form of a network and empirically explores the critical and controllable risk sources, thereby contributing to the risk management practice of CICCs in Africa. Notably, although we focus on Chinese employees in Africa, international construction companies in other countries can also apply the approach adopted in this study when formulating safety risk prevention measures for their employees.

A limitation of this study concerns the representativeness of the data. Due to the lack of a sampling frame, we adopted a non-probability sampling method. This resulted in a high number of questionnaires from Central Africa and a relatively low number of questionnaires from West Africa. In addition, this paper is mainly based on Heinrich’s Domino Theory of Accident Causation, which conceptualizes the risk relationship as “risk sources → risk events → risk consequences” without considering the direct relationship between risk source and risk consequence. This may lead to an underestimation of the role of uncontrollable original risk sources, which were also not explored in this research. Future research can focus on these uncontrollable safety risk sources and their impact on safety risk consequences.

## Figures and Tables

**Table 1:** Results of non-traditional safety risk factors of employees from CICCs in Africa.

Category		Variables	Reference
Risk sources	Uncontrollable original political risk sources (UPRS)	Outbreak of war	Case 4; [16,42]
		Political unrest	Case 1, 8; [16,57]
		Government Corruption	[58,61]
	Subsequent political risk sources (SPRS)	Loss of protection from the host country	[57]
		Collusion between police and bandits	[17,62]
		Denial of justice	Case 7; [58]
	Uncontrollable original economic risk sources (UECRS)	Loss or bankruptcy of the owner of the project	[63]
		Financial conflicts of interest between China and the host country	Case 3, 6
		Economic deterioration	Case 10, 15; [15,42]
	Subsequent economic risk sources (SECRS)	The failure to pay salaries to Chinese employees	[15,64]
		Labor disputes	Case 3, 6
	Uncontrollable original sociocultural risk sources (USRS)	Religious, ethnic, and tribal conflicts	[14,57]
		Cultural differences	[16]
		Social class conflicts	[57]
		Public security disorder	Case 4, 11; [15,58]
	Subsequent sociocultural risk sources (SSRS)	Two-way racism between Chinese employees and locals	Case 13; [65]
		Being marginalized or discriminated	[18]
	Uncontrollable original environmental risk sources (UENRS)	Poor living conditions (Catering, residential, medical, etc.)	Case 9; [62]
		Construction sites are located in remote areas	Case 5, 8, 11
		Outbreaks of infectious diseases	Case 9, 12; [15,17]
	Subsequent environmental risk sources (SENRS)	The construction area is exposed to an unsafe environment	Case 2, 4, 5
	Controllable behavioral risk sources (CBRS)	Unlawful activities	[61]
		Lack of safety awareness	[58]
		Do not respect local customs	[65]
		Damage the local environment	Case 10; [62,66]
		Do not provide employments for locals	Case 10; [59,65]
		Differential treatment between local and Chinese employees	[59,65]
Risk events	Armed conflicts (AC)	Explosion	Case 2, 5; [24]
		Shooting	Case 8, 14; [24,66]
	Criminal offense (CO)	Robbery	Case 13, 15; [14,24]
		Theft	[14,17]
		Kidnapping	Case 1, 11; [18,67]
		Fraud	Case 7; [68]
		Riot	Case 6, 10; [17,62]
	Disease and accidental injury (DAI)	Getting into an automobile accident	[68]
		Exposure to natural disasters	[63]
		Suffering from an infectious disease	[15,62]
Risk consequences	Safety risk consequences (SRC)	Casualties	Case 3, 8; [17,18,67]
		Property loss	Case 11, 14; [17]

**Table 2:** The basic information of the respondents.

Feature	Category	Frequency	Percentage
Type of company	General contractor	95	45.67%
	Subcontractor	95	45.67%
	Other	18	8.65%
Position	Senior manager	31	14.90%
	Manager	52	25.00%
	Supervisor	47	22.60%
	Professional engineer	42	20.19%
	Construction worker	25	12.02%
	Other	11	5.29%
Years of work	Less than 2 years	84	40.38%
	2-5 years	62	29.81%
	5-10 years	43	20.67%
	10-15 years	14	6.73%
	Over 15 years	5	2.40%
Work region	North Africa	16	7.69%
	East Africa	28	13.46%
	West Africa	13	6.25%
	Central Africa	117	56.25%
	South Africa	34	16.35%

**Table 3:** Results of internal reliability and Confirmatory factor analysis (CFA).

Latent Variables	Observed Variables	Mean	S.D.	Factor Loading	Cronbach’s Alpha	AVE	CR	BSIBLV
Uncontrollable original political risk sources (UPRS)	Outbreak of war	2.75	1.127	0.754	0.702	0.546	0.782	0.654^2^ < 0.546
	Political unrest	3.2	1.052	0.677				
	Government Corruption	4.02	0.971	0.781				
Subsequent political risk sources (SPRS)	Loss of protection from host country	2.88	1.026	0.732	0.839	0.639	0.841	0.658^2^ < 0.639
	Collusion between police and bandits	3.27	1.152	0.844				
	Denial of justice	3.28	1.061	0.817				
Uncontrollable original economic risk sources (UECRS)	Loss or bankruptcy of the owner of the project	3.11	1.098	0.776	0.758	0.527	0.768	0.720^2^ < 0.527
	Financial conflicts of interest between China and the host country	2.67	1.014	0.622				
	Economic deterioration	3.48	1.083	0.769				
Subsequent economic risk sources (SECRS)	The failure to pay salaries to Chinese employees	2.94	1.216	0.724	0.704	0.558	0.716	0.692^2^ < 0.558
	Labor disputes	2.95	0.982	0.769				
Uncontrollable original sociocultural risk sources (USRS)	Religious, ethnic and tribal conflicts	2.82	1.034	0.784	0.794	0.534	0.818	0.649^2^ < 0.534
	Cultural differences	3.35	0.971	0.598				
	Social class conflicts	3.14	1.041	0.668				
	Public security disorder	3.27	1.122	0.847				
Subsequent sociocultural risk sources (SSRS)	Demonstration, parade	3.16	1.092	0.727	0.712	0.516	0.68	0.520^2^ < 0.516
	Being marginalized or discriminated	2.56	1.004	0.709				
Uncontrollable original environmental risk sources (UENRS)	Poor living conditions (Catering, residential, medical, etc.)	3.4	1.034	0.74	0.83	0.629	0.836	0.758^2^ < 0.629
	Construction sites are located in remote areas	3.22	1.088	0.833				
	Outbreaks of infectious diseases	2.76	0.971	0.804				
Subsequent environmental risk sources (SENRS)	The construction area is exposed to an unsafe environment	2.92	1.02	1	/	/		/
(Controllable) behavioral risk sources (CBRS)	Unlawful activities	2.24	0.897	0.628	0.857	0.514	0.858	0.652^2^ < 0.514
	Lack of safety awareness	2.53	1.028	0.808				
	Do not respect local customs	2.3	1.041	0.896				
	Damage the local environment	2.27	1.058	0.822				
	Do not provide employments for locals	2.17	1.042	0.545				
	Differential treatment between local and Chinese employees	2.73	1.025	0.508				
Armed conflicts (AC)	Explosion	2.16	1.092	0.834	0.853	0.749	0.857	0.649^2^ < 0.749
	Shooting	2.32	1.218	0.896				
Criminal offense (CO)	Robbery	3.39	1.011	0.68	0.779	0.513	0.84	0.652^2^ < 0.513
	Theft	3.83	1.096	0.679				
	Kidnapping	2.38	1.065	0.819				
	Fraud	2.66	1.173	0.697				
	Riot	2.94	1.158	0.698				
Disease and accidental injury (DAI)	Exposure to natural disasters	2.47	0.934	0.69	0.703	0.532	0.694	0.673^2^ < 0.532
	Suffering from an infectious disease	3.33	1.058	0.767				
Safety risk consequences (SRC)	Casualties	2.6	1.045	0.895	0.832	0.718	0.836	0.684^2^ < 0.718
	Property loss	2.97	0.999	0.797				

Model fit: CFI = 0.894, RMSEA = 0.063, PNFI = 0.718, CMIN/DF = 1.922.

**Table 4:** Significant safety risk paths and path coefficients.

Path	Uncontrollable Risk Sources	Coefficient	Subsequent Risk Sources	Coefficient	Risk Events	Coefficient	Risk Consequences	Path Coefficient
1	UPRS	0.237	SPRS	0.249	CO	0.62	SRC	0.037
		→		→		→		
2	UECRS	0.696	SPRS	0.249	CO	0.62	SRC	0.107
		→		→		→		
3	USRS	0.674	SPRS	0.249	CO	0.62	SRC	0.104
		→		→		→		
4	UECRS	0.853	SECRS	0.204	CO	0.62	SRC	0.108
		→		→		→		
5	UENRS	0.51	SECRS	0.204	CO	0.62	SRC	0.065
		→		→		→		
6	UECRS	0.55	SSRS	0.365	CO	0.62	SRC	0.124
		→		→		→		
7	USRS	0.826	SSRS	0.365	CO	0.62	SRC	0.187
		→		→		→		
8	UPRS	0.276	SENRS	0.848	DAI	0.398	SRC	0.093
		→		→		→		
9	USRS	0.233	SENRS	0.848	DAI	0.398	SRC	0.079
		→		→		→		
10	UENRS	0.932	SENRS	0.848	DAI	0.398	SRC	0.315
		→		→		→		
**Path**	**Controllable Risk Sources**			**Coefficient**	**Risk Events**	**Coefficient**	**Risk Consequences**	**Path Coefficient**
11	CBRS			0.623	CO	0.62	SRC	0.386
				→		→		
12	CBRS			0.16	DAI	0.398	SRC	0.064
				→		→		

Model fit: CFI = 0.905; RMSEA = 0.051; PNFI = 0.722; CMIN/DF = 2.341.Abbreviation Description: Uncontrollable original political risk sources (UPRS); Uncontrollable original economic risk sources (UECRS); Uncontrollable original sociocultural risk sources (USRS); Uncontrollable original environmental risk sources (UENRS); (Controllable) behavioral risk sources (CBRS); Subsequent political risk sources (SPRS); Subsequent economic risk sources (SECRS); Subsequent sociocultural risk sources (SSRS); Subsequent environmental risk sources (SENRS); Criminal offense (CO); Disease and accidental injury (DAI); Safety risk consequences (SRC).

**Table 5:** Total effects of controllable risk sources.

Controllable and Subsequent Risk Sources	CBRS	SPRS	SECRS	SSRS	SENRS
Total effects	0.347 ***	0.154 *	0.126 *	0.226 **	0.338 ***

Significant level: *** *p* < 0.01; ** *p* < 0.05; * *p* < 0.1. Abbreviation Description: (Controllable) behavioral risk sources (CBRS); Subsequent political risk sources (SPRS); Subsequent economic risk sources (SECRS); Subsequent sociocultural risk sources (SSRS); Subsequent environmental risk sources (SENRS).

## Data Availability

The data presented in this study are available on request from the corresponding author. The data are not publicly available due to privacy and confidentiality issues.

## Appendix A

**Table A1:** Case descriptions.

No.	Year	Country	Case Descriptions
1	2007	Ethiopia	An attack by unidentified militants on a construction site in Ethiopia by the China National Crude Oil Field Exploration Bureau (CNREDB) has resulted in the killing of 74 people, including nine Chinese workers, and the taking of seven others into captivity. The militants had withdrawn by the time a brigade sent by Ethiopia to reinforce them arrived at the site.
2	2007	Algeria	Two explosions occurred in the Algerian capital, the first in the new office building of the Algerian Constitutional Council, built by China State Construction Engineering Corporation (CSCEC), and the second in the Hydra area of Algiers province. In the first explosion, one employee from CSCEC was tragically killed and seven employees were injured.
3	2008	Equatorial Guinea	In a Chinese construction site in Equatorial Guinea, more than 400 Chinese workers went on strike after failing to defend their rights and interests over a labor dispute, resulting in a clash with local police that left two Chinese workers dead and four injured.
4	2011	Libya	The outbreak of war in Libya has led to a sharp deterioration in the social security situation, with violent incidents such as vandalism and looting continuing to occur. According to the relevant statistics, more than ten employees of Chinese enterprises were injured and their sites and camps were attacked and robbed, resulting in direct economic losses of RMB 1.5 billion.
5	2012	The Republic of Congo	An explosion at an ammunition depot in Brazzaville, the capital of the Republic of Congo, has affected the nearby Beijing Construction Group project site, killing six Chinese employees and injuring 45 others to varying degrees.
6	2012	Zambia	Local workers protested against the delayed implementation of the new minimum wage in the Chinese coal mine, which in turn led to mass riots, culminating in the death of one Chinese manager and the injury of four Chinese workers, and ultimately forced the closure of the mine.
7	2013	Chad	Several cases of Chinese nationals being blackmailed in the Chadian capital N’Djamena, with some unscrupulous police officers using security checks as a pretext to take the Chinese people involved to the police station by unorthodox means, extort money and then release them.
8	2014	Cameroon	Unidentified militants attacked the camp of the 16th Engineering Bureau of China Water Resources and Hydropower Corporation in the northern region of Cameroon. A Chinese employee was hit by a stray bullet. The other 10 people lost contact and 10 vehicles in the camp were robbed.
9	2015	Mali	Al-Qaeda took 170 hostages in a hotel in Bamako, Mali. Three China Railway Construction Group Corporation (CRCGC) executives were killed, who planned to discuss cooperation projects with the Ministry of Transport in Mali.
10	2016	Kenya	In a Chinese-funded railway project in Narok County, southwest Kenya, the Kenyan government was accused of not paying attention to the employment of local youths in the railway project, and some 200 local youths expressed their dissatisfaction at not being able to access employment opportunities in the railway project and injured 14 Chinese workers.
11	2017	Nigeria	A Chinese worker was kidnapped in the far suburbs of Abuja, Nigeria. The kidnappers broke into the factory of a Chinese company at gunpoint and took a Chinese worker captive.
12	2017	Kenya	According to Kenyan media reports, as of July 18, there were 150 confirmed cases of Dengue Fever in Ken Mombasa and 336 confirmed cases of Cholera in Nairobi.
13	2018	Zambia	A small-scale riot and demonstration against the Chinese in Kitwe, Copperbelt Province, Zambia, resulted in several Chinese shops and businesses being robbed and suffering more serious material and property losses. The Chinese Embassy initially concluded that the main reason was that some locals had heard rumors that “the government had sold the Zambian forestry company to the Chinese”.
14	2019	The Democratic Republic of the Congo	More than a dozen armed men in Ituri province attacked a Chinese mining site in the Mambasa district of the province. The militants burned vehicles, looted valuables, and killed three staff members, including two Chinese employees.
15	2020	Tanzania	During the outbreak of Covid-19, security risks and instability in Zanzibar rose further due to an increase in the number of unemployed people. An incident of theft and robbery at a Chinese enterprise camp in Zanzibar resulted in two Chinese employees sustaining minor injuries and a certain amount of property loss.

Sources: The Chinese Ministry of Commerce’s “Go Global Public Service Platform”, The China Consular Service Network.
